# Monte Carlo calculations and experimental measurements of the TG‐43U1‐recommended dosimetric parameters of ^125^I (Model IR‐Seed2) brachytherapy source

**DOI:** 10.1120/jacmp.v17i4.6127

**Published:** 2016-07-08

**Authors:** Sahar Sheikholeslami, Hassan Ali Nedaie, Mahdi Sadeghi, Hosein Pourbeigy, Sohrab Shahzadi, Mehdi Zehtabian, Mohsen Hasani, Ali S. Meigooni

**Affiliations:** ^1^ Department of Engineering Science and Research Branch, Islamic Azad University Tehran Iran; ^2^ Department of Radiotherapy Oncology Radiotherapy & Radiobiology Research Centre, Cancer Institute, Tehran University of Medical Sciences Tehran Iran; ^3^ Radiation Application Research School, Nuclear Science and Technology Research Institute Tehran Iran; ^4^ Applied Radiation Research School, Nuclear Science and Technology Research Institute Tehran Iran; ^5^ Shohada Medical Center, Shaheed Beheshti University of Medical Sciences Tehran Iran; ^6^ Nuclear Engineering Department Shiraz University Shiraz Iran; ^7^ Comprehensive Cancer Centers of Nevada Las Vegas Nevada USA

**Keywords:** I125 (Model IR‐Seed2), brachytherapy, Monte Carlo simulation, thermoluminescent dosimeter, TG‐43U1

## Abstract

A new design of I125 (Model IR‐Seed2) brachytherapy source has been manufactured recently at the Applied Radiation Research School, Nuclear Science and Technology Research Institute in Iran. The source consists of six resin beads (0.5 mm diameter) that are sealed in a cylindrical titanium capsule of 0.7 mm internal and 0.8 mm external diameters. This work aims to evaluate the dosimetric parameters of the newly designed I125 source using experimental measurements and Monte Carlo (MC) simulations. Dosimetric characteristics (dose rate constant, radial dose function, and 2D and 1D anisotropy functions) of the IR‐Seed2 were determined using experimental measurements and MC simulations following the recommendations by the Task Group 43 (TG‐43U1) report of the American Association of Physicists in Medicine (AAPM). MC simulations were performed using the MCNP5 code in water and Plexiglas, and experimental measurements were carried out using thermoluminescent dosimeters (TLD‐GR207A) in Plexiglas phantoms. The measured dose to water in Plexiglas data were used for verification of the accuracy of the source and phantom geometry in the Monte Carlo simulations. The final MC simulated data to water in water were recommended for clinical applications. The MC calculated dose rate constant (Λ) of the IR‐Seed2 I125 seed in water was found to be $0.992\pm 0.025\;\text{cGy}\;h^{-1}U^{-1}$. Additionally, its radial dose function by line and point source approximations, $g_{L}(r)$ and $g_{p}(r)$, calculated for distances from 0.1 cm to 7 cm. The values of $g_{L}(r)$ at radial distances from 0.5 cm to 5 cm were measured in a Plexiglas phantom to be between 1.212 and 0.413. The calculated and measured of values for 2D anisotropy function, F(r,θ), were obtained for the radial distances ranging from 1.5 cm to 5 cm and angular range of 0°‐90° in a Plexiglas phantom. Also, the 2D anisotropy function was calculated in water for the clinical application. The results of these investigations show that the uncertainty of the experimental data is within ±7% between the measured and simulated data in Plexiglas. Based on these results, the MC‐simulated dosimetric parameters of the new I125 source model in water are presented for its clinical applications in brachytherapy treatments.

PACS number(s): 87.56.bg

## I. INTRODUCTION

I125 and P103d brachytherapy seeds share a great role in brachytherapy implants in various tumor sites such as the eye and prostate tumors.^(1)^ In addition to the strong inverse‐square law reduction in gamma ray fluence at short distances, low‐energy photon emissions of I125 sources lead to a rapid decrease in radiation dose with increasing distance. Therefore, this effect reduces the unnecessary radiation dose to normal tissue located beyond the tumor. Different designs of I125 sources, such as IsoAid Advantage (IsoAid, LLC, Port Richey, FL), BEBIG model 125. So6 (Eckert & Ziegler BEBIG Inc., Mount Vernon, NY), and Best Medical model 2301 (Best Medical International, Springfield, VA) are commercially available.^2^, ^3^ Recently, a new design of I125 (Model IR‐Seed2) brachytherapy seed has been produced for clinical applications by the Applied Radiation Research School, Nuclear Science and Technology Research Institute, Tehran, Iran. TG‐43U1 recommends that the dosimetric parameters of each new source model must be determined before its clinical application.^(3)^

The purpose of this study is to determine the TG‐43U1 recommended dosimetric parameters of the Model IR‐Seed2 I125 source using Monte Carlo (MC) simulation and experimental measurements. The MC simulations were performed using the MCNP5 Monte Carlo code in water and Plexiglas phantom materials. The experimental procedures were performed by using TLD chips in Plexiglas phantoms. The results of these investigations were compared with the published data of other commercially available I125 sources.^4^, ^5^

## II. MATERIALS AND METHODS

### A. IR‐Seed2 I125 source

**Figure 1:**
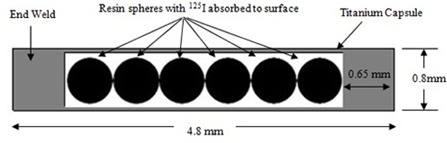
Schematic diagram of the Model IR‐Seed2 I125 brachytherapy source.

Figure 1 shows a schematic diagram of the IR‐Seed2 I125 source. This source model consists of six spherical beads with percentage weight composition of: H, 8%; C, 90%; N, 0.3%; Cl, 0.7%; I, 1%. To access high activity up to 40 mCi, the IR‐Seed2 seed has been manufactured without X‐ray marker. Diameter of each bead is 0.5 mm and the beads are sealed within a titanium cylindrical tube with external diameter of 0.8 mm. The two ends of this tube are sealed using 0.65 mm thick titanium by a laser welding technique which is seen in radiography/CT images. The physical length inside would be=2*0.65+6*0.5+0.5 mm; gap between the pellets=4.8 mm; and outer diameter of the source is 0.8 mm. The effective active length of the source is 3.6mm(6×0.6 mm spaces between the beads).

The I125 radioactive material is uniformly adsorbed on the surface of each resin bead. The activities of the beads within the source are distributed in a symmetrical fashion and they are arranged as follows: each of the outermost beads were 2.2 mCi, the next two beads were 2.3 mCi, and the remaining two beads (closest to the center of the source) were 1.7 mCi.

### B. TG‐43U1 dose calculation formalism

Characteristics of the Model IR‐Seed2 source were determined according to the recommendations of the Task Group 43 (TG‐43U1) by the American Association of Physicists in Medicine (AAPM).^3^, ^4^ Following this protocol, the spatial dose rate distribution $\dot{D}(r,\theta )$ around a sealed brachytherapy source can be determined using the following formula:

(1) D˙(r,θ)=ΛSKG(r,θ)G(1 cm,π2)gL(r)F(r,θ),

where Λ is the dose rate constant at a reference point of (1 cm,π2),SK is the air kerma strength of the source, G(r,θ) is the geometry function, $g_{L}(r)$ is the line radial dose function, and F(r,θ) is the 2D anisotropy function. The above quantities are discussed in detail in the AAPM TG‐43 report.^(4)^

The air kerma strength, $S_{K}$, was calculated using the recommended equation below:

(2) $$S_{K}={\dot{K}}_{\delta }(r)r^{2}$$

Due to the low energy of the photons from I125 and small range of secondary electrons produced by photons emitted from the source, it was assumed in the Monte Carlo calculations that all electrons generated by the photon collisions are absorbed locally, so it was assumed that dose is equal to kerma at all points of interest.

### C. Thermoluminescent dosimetry

The dose distribution around the source was measured to water in Plexiglas phantoms using TLD GR207A chips with dimensions of 4.5 mm×0.8 mm. These chips are also known as 7‐LiF:Mg,Cu,P thermoluminescent dosimeters (Fimel, Vélizy, France). These chips were annealed following the procedure recommended by the manufacturer (240°C, 10 min).^(6)^

In order to reduce the effect of statistical fluctuations in the measured data, the averages from several TLD chips were used as the representative of the data for a given point. However, the differences of the mass and physical geometries of the TLD chips may lead to some variation in their responses. In order to practically eliminate these effects, a correction factor was introduced for each TLD chip by calculating the ratio of the measured responses of the individual chip to the average response from the entire batch, when all were exposed to the same dose. Each correction factor is referred to as the element correction coefficient (ECC).

#### C.1 Calibration

**Figure 2:**
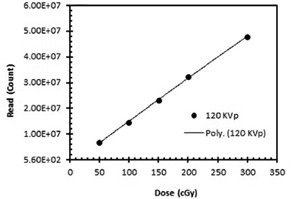
TLD calibration curve with an 120 kVp X‐ray beam.

The ECCs of the TLDs and the dose‐response curve were acquired via irradiation by 6 MV and 120 kVp X‐ray beams, respectively. The dose‐response curve was obtained using doses ranging from 50 cGy to 300 cGy. This dose range was selected to cover the dose range that was used in this research project. However, the TLD calibration factor (ε: response per cGy) was determined using a 120 kVp X‐ray beam. Figure 2 displays the TLD dose‐response curve.

The irradiated TLDs were read out by an LTM TLD reader (Fimel, Vélizy, France). The following equation was used to calculate the dose rate per air‐kerma strength from the TLD responses for each point irradiated in the phantom:

(3) $$\frac{\dot{D}(r,\theta )}{S_{K}}=\frac{R}{TS_{K}\varepsilon E(r)d(T)F_{lin}},$$

where $\dot{D}(r,\theta )$ was the absorbed dose rate at the start of the irradiation at a point (*r*, θ)*, R* was the TLD response that had been corrected for physical differences between the TLD chips and the background using the predetermined chip factors,^1^, ^7^, ^8^ *T* was the irradiation time (hours), and ε was the calibration factor for the TLD response (nC/cGy). To obtain the calibration factor, 18 TLD chips were placed in a custom‐designed Plexiglas slab phantom in a kilovoltage X‐ray (120 kVp) field that was calibrated using the TRS 398 protocol of the International Atomic Energy Agency (IAEA).^(9)^ E(r) was the correction factor for the energy dependence of the TLDs between the calibration beam and the I125 photons, which is equal to unity in this study.^(10)^ The parameter d(T) is a correction factor which was used to account for source decay during the exposure.^(7)^ $F_{\text{lin}}$ is the nonlinearity correction factor of the TLD response for the given dose. Responses of the TLDs were linear within the range of the doses used in this project.

**Figure 3:**
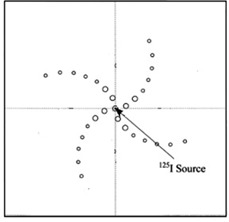
Schematic diagram of the experimental setup for measurement of radial dose function.

**Figure 4:**
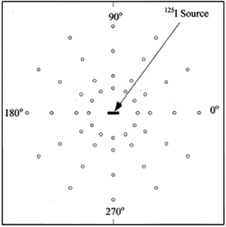
Schematic diagram of the experimental setup for measurement of anisotropy function.

Two Plexiglas phantoms with dimensions of $30\times 30\times 15\;{\text{cm}}^{3}$ were used to measure radial dose function g(r), dose rate constant (Λ) and 2D anisotropy function of the I125 source.^(11)^ The thickness of each phantom was 1 cm. The overall phantom setup for the experimental procedures were composed of 15 slabs of Plexiglas. The central slab for each phantom was machined to house the TLDs. The experimental setup for measurement of the anisotropy function and radial dose function are shown in Figs. 3 and 4. The error propagation of the experimental data is shown in Table 1. The seed geometry error is due to differences of air gap distance between spheres and resin bead diameters in Monte Carlo simulation and reality.

**Table 1:** Uncertainty determination for the experimental measurements using TLDs and Monte Carlo simulation

*Component*	*TLD Uncertainties*	
	*Type A*	*Type B*
Repetitive TLD measurements	4.4%	
TLD dose calibration		1.41%
Source to TLD position		1.1%
Correction of energy dependence of LiF		5.5%
Quadrature combination	4.4%	5.8%
Total combined standard uncertainty $\left(\mu_{c}\right)$	7.2%	
	*Monte Carlo Uncertainties*	
Component	r=1 cm	r=5 cm
Statistics	0.05%	0.07%
Photoionization^a(3)^	1.5%	4.5%
Cross‐section (2.3%)^(3)^		
Seed geometry(3)	2.0%	2.0%
Source energy spectrum^a(3)^	0.1%	0.3%
Quadrature sum	2.5%	4.5%

a ^a^ On the transverse plane.

### D. Monte Carlo calculations

Version 5 of the MCNP code developed by Los Alamos National Laboratory (Los Alamos, NM) was used to perform the simulations for these investigations.^(12)^ There are several different tally types available in the MCNP5 code for scoring diverse physical characteristics.^(12)^*F4 tally was used to determine the energy flux in MeV/cm^2^, which could be converted to absorbed dose by applying suitable r=1 cm coefficients. The I125 photon spectrum and photoionization in this simulation were extracted from TG‐43U1 report. In these calculations, the titanium characteristic X‐ray production was suppressed with the energy cutoff δ=5 keV.^(13)^ In the Monte Carlo calculation, according to the cross‐section data in the Monte Carlo library, the effect of self‐adsorptions has been automatically considered. Simulations were performed to calculate absorbed dose to water in Plexiglas in order to provide data comparable with the TLD measurements. Once the Monte Carlo calculations in Plexiglas were shown to be in agreement with the TLD data (within experimental uncertainty), the calculations were performed to water in water to obtain data for clinical applications per TG‐43 protocol.

A spherical water phantom of 30 cm diameter (with an atomic ratio of 2:1 for H:O and $\rho =0.998\frac{g}{{\text{cm}}^{3}}$) was modeled. The phantom size in this simulation was comparable with the experimental setup (i.e., 30 cm×30 cm×15 cm). The composition of the Plexiglas was H, 8%; C, 60%; and O, 32%, with a mass density of 1.19 g/cm^3^.^(14)^ To calculate its dosimetric parameters, the seed was simulated in the center of the phantom and the simulations were performed for radial distances of *r* = 0.1, 0.2, …, 7 cm away from the source and at polar angles relative to the longitudinal axis of the seed from 0° to 90° with 5° increments.

## III. RESULTS

### A. Dose rate constant

The dose rate constant, Λ, of the source calculated in water was found to be $0.992\pm 0.025\;\text{cGy}\;U^{-1}h^{-1}$ (Table 2). Table 2 also presents a comparison of dose rate constant obtained for this source model with those for the other commercially available brachytherapy sources.

**Table 2:** Comparison of the calculated dose rate constant, Λ, obtained in this study and some of those reported in the literature

*Source Type*	*Method*	*Medium*	*Dose‐Rate Constant* $\Lambda (cGy.h^{-1}.U^{-1})$
IR‐Seed2	Monte Carlo (MCNP5)	Liquid water	0.992±0.025
Amersham 6702^(3)^	Monte Carlo	Liquid water	1.036
IBt 1251L^(16)^	Monte Carlo	Liquid water	1.038
MBI SL‐125/SH‐125^(16)^	Monte Carlo	Liquid water	0.953

### B. Radial dose function

**Figure 5:**
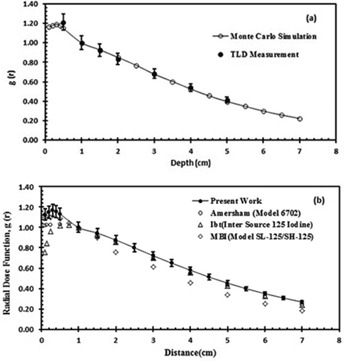
A comparison (a) between the measured and calculated radial dose function of the Model IR‐Seed2 I125 source in a Plexiglas phantom. The solid line represents the fifth‐order polynominal fit to the data of the present study. The error bars represent ±7%; (b) shows a comparison of the calculated radial dose function of the IR‐Seed2 source in water with three other brachytherapy sources. The line represents the fifth‐order polynominal fit to the data of the present study. The error bars represent ±7%.

Figure 5(a) shows a comparison between the calculated and measured radial dose functions $g_{L}(r)$ of the IR‐Seed2 in a Plexiglas phantom. The uncertainties of the measured and calculated data were ±7% and ±7%, respectively. A good agreement (within experimental uncertainties) between the measured and calculated values can be seen in this figure. Figure 5(b) shows a comparison between the calculated $g_{L}(r)$ of the Model IR‐Seed2 seed with three other available brachytherapy sources.^3^, ^15^ The values of $g_{L}(r)$ and $g_{P}(r)$ in Plexiglas and water phantoms are listed in Table 3.

**Table 3:** Calculated and measurement radial dose function, $g_{L}(r)$ and $g_{P}(r)$ values for line and point‐source approximation, of the IR‐Seed2 brachytherapy source in Plexiglas and water

*Distance From Source Center r (cm)*	$g_{L}(r)$ *Measurement (Plexiglas)*	$g_{L}(r)$ *Monte Carlo (Plexiglas)*	$g_{L}(r)$ *Monte Carlo (Water)*	^g^ *P^(r)^Monte Carlo (Water)*
0.1	**‐**	1.164	1.126	0.900
0.2	**‐**	1.176	1.148	1.010
0.3	**‐**	1.192	1.167	1.085
0.4	**‐**	1.178	1.157	1.082
0.5	1.212	1.149	1.132	1.094
1	1.000	1.000	1.000	1.000
1.5	0.923	0.929	0.945	0.950
2	0.833	0.851	0.876	0.883
2.5	**‐**	0.770	0.799	0.806
3	0.682	0.679	0.722	0.729
3.5	**‐**	0.604	0.648	0.655
4	0.543	0.527	0.579	0.585
4.5	**‐**	0.457	0.512	0.517
5	0.413	0.399	0.452	0.457
5.5	**‐**	0.349	0.398	0.402
6	**‐**	0.300	0.349	0.353
6.5	**‐**	0.260	0.306	0.309
7	**‐**	0.225	0.267	0.270

The measurements indicate up to 5% differences in radial dose function values for the IR‐Seed2 seed at distance of 0.5 cm compared to the MC simulated data due to the high dose gradient. The radial dose function in water for clinical application was fitted to a fifth order polynomial function as follows:

(4) $$g_{L}(r)=a_{0}+a_{1}r^{1}+a_{2}r^{2}+a_{3}r^{3}+a_{4}r^{4}+a_{5}r^{5}$$

where $a_{0}=1.1731,\;a_{1}=-9.1348\times 10^{-2},\;a_{2}=-6.2446\times 10^{-2},\;a_{3}=2.2023\times 10^{-2},\;a_{4}=-3.1701\times 10^{-3},\;\text{and}\;a_{5}=1.6987\times 10^{-4}$.

### C. Anisotropy function

**Figure 6:**
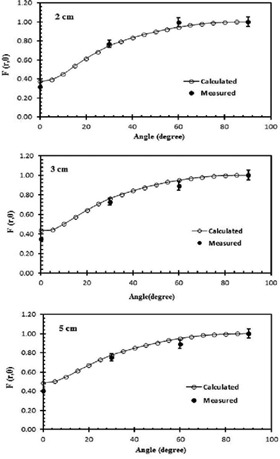
A comparison of the measured and calculated anisotropy function of the IR‐Seed2 source in a Plexiglas phantom at 2 cm, 3 cm, and 5 cm distances. The line show a 4th polynominal fit to the data.

**Figure 7:**
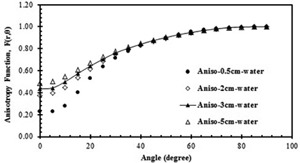
The variation of the calculated anisotropy function of the Model IR‐Seed2 I125 source in water at distances ranging from 0.5 cm to 5 cm.

The anisotropy function, F(r,θ), of the IR‐Seed2 was measured and calculated at 30° and 5° intervals, respectively, at radial distances of 1.5, 2, 3, and 5 cm relative to the center of the seed and polar angles (θ) ranging from 0° to 90°. The results can be seen in Table 4. Table 5 shows the MC‐calculated data in water ranging from 0.5 cm to 7 cm. Figure 6 shows a comparison between the calculated and measured F(r,θ) at various angles in the Plexiglas phantom. The uncertainties of the measured and calculated data were ±7% and ±7%, respectively. Because of the limited size of the TLD‐GR207A chips and the uncertainty of the experiments during the multifold TLD measurements, this large variation in dose cannot be fully tracked. Figure 7 presents the variations in the calculated F(r,θ) in water at various distances.

**Figure 8:**
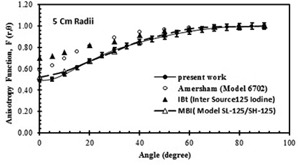
Comparison of the MC calculated anisotropy functions of the Model IR‐Seed2 I125 source in water with the three other brachytherapy sources. The error bars on the data for the IR‐Seed2 source are ±7%.

A comparison between the calculated anisotropy function of the new source at 5 cm radii from the axis of the seed in water with previously published data for three other brachytherapy sources is shown in Fig. 8.^(15)^

**Table 4:** Measured and calculated anisotropy function values of the Model IR‐Seed2 I125 source in a Plexiglas phantom

*Angle* θ *(degrees)*	*Measured* F(r,θ)				*Calculated* F(r,θ)			
	r=1.5 cm	r=2 cm	r=3 cm	r=5 cm	r=1.5 cm	*r=2 cm*	*r=3 cm*	r=5 cm
0	0.282	0.319	0.348	0.400	0.344	0.373	0.435	0.487
5	**‐**	**‐**	**‐**	**‐**	0.355	0.394	0.443	0.502
10	**‐**	**‐**	**‐**		0.417	0.449	0.499	0.548
15	**‐**	**‐**	**‐**	**‐**	0.507	0.535	0.573	0.610
20	**‐**	**‐**	**‐**	‐	0.595	0.614	0.642	0.671
25	**‐**	**‐**	**‐**	**‐**	0.670	0.685	0.705	0.726
30	0.757	0.767	0.726	0.751	0.738	0.748	0.761	0.776
35	**‐**	**‐**	**‐**	**‐**	0.787	0.792	0.803	0.813
40	**‐**	**‐**	**‐**	‐	0.829	0.833	0.840	0.847
45	**‐**	**‐**	**‐**	**‐**	0.862	0.866	0.874	0.877
50	**‐**	**‐**	**‐**	‐	0.894	0.896	0.901	0.904
55	**‐**	**‐**	**‐**	**‐**	0.921	0.922	0.928	0.929
60	0.894	0.994	0.891	0.892	0.946	0.944	0.947	0.948
65	**‐**	**‐**	**‐**	**‐**	0.965	0.964	0.966	0.967
70	**‐**	**‐**	**‐**	‐	0.981	0.978	0.980	0.980
75	**‐**	**‐**	**‐**	**‐**	0.991	0.989	0.992	0.990
80	**‐**	**‐**	**‐**	‐	0.998	0.995	0.997	0.994
85	**‐**	**‐**	**‐**	**‐**	0.999	0.998	0.999	0.999
90	1.000	1.000	1.000	1.000	1.000	1.000	1.000	1.000
$\emptyset_{\text{an}}(r)$	0.857	0.849	0.841	0.863	0.859	0.860	0.858	0.878

**Table 5:** MC‐calculated anisotropy function values of the Model IR‐Seed2 I125 brachytherapy source in water

*Angle (degrees)*	*0.5 cm*	*1 cm*	*1.5 cm*	*2 cm*	*3 cm*	*4 cm*	*5 cm*	*7 cm*
0	0.230	0.289	0.344	0.373	0.435	0.473	0.487	0.505
5	0.229	0.305	0.355	0.394	0.443	0.476	0.502	0.531
10	0.277	0.366	0.417	0.449	0.499	0.523	0.548	0.570
15	0.403	0.466	0.507	0.535	0.573	0.590	0.610	0.630
20	0.529	0.566	0.595	0.614	0.642	0.658	0.671	0.684
25	0.636	0.651	0.670	0.685	0.705	0.714	0.726	0.737
30	0.716	0.725	0.738	0.748	0.761	0.764	0.776	0.780
35	0.776	0.778	0.787	0.792	0.803	0.807	0.813	0.818
40	0.825	0.822	0.829	0.833	0.840	0.842	0.847	0.850
45	0.865	0.858	0.862	0.866	0.874	0.871	0.877	0.881
50	0.899	0.891	0.894	0.896	0.901	0.899	0.904	0.906
55	0.929	0.921	0.921	0.922	0.928	0.921	0.929	0.927
60	0.952	0.945	0.946	0.944	0.947	0.942	0.948	0.945
65	0.971	0.966	0.965	0.964	0.966	0.964	0.967	0.968
70	0.982	0.981	0.981	0.978	0.980	0.978	0.980	0.981
75	0.990	0.992	0.991	0.989	0.992	0.985	0.990	0.988
80	0.995	0.997	0.998	0.995	0.997	0.992	0.994	0.995
85	0.999	0.999	0.999	0.998	0.999	0.992	0.999	0.997
90	1.000	1.000	1.000	1.000	1.000	1.000	1.000	1.000
$\emptyset_{an}(r)$	0.898	0.849	0.844	0.850	0.856	0.857	0.860	0.862

## IV. DISCUSSION

This study was performed to evaluate the dosimetric parameters of a newly designed I125 brachytherapy source (IR‐Seed2) using TLD measurements and MC calculations in water and Plexiglas phantoms. These determinations were performed in accordance with the TG‐43U1 recommendations.^(3)^ The results of these investigations were compared with the published data by the Task Group 43 report.^3^, ^15^ The value of the dose rate constant, Λ, in water was calculated for clinical applications and was found to be $0.992\pm 0.025\;\text{cGy}\;U^{-1}h^{-1}$, which is close to the dose‐rate constant of I125 source, for 6702,^(3)^ MBI,^(15)^ and IBt^(15)^ models. The calculated dosimetric parameters of the source are within ±7% of the published data for those brachytherapy sources. In this project, high precision was obtained in the results of the simulations, due to simulating large numbers of histories (up to $1.1\times 10^{9}$) and using variance reduction methods.

The radial dose function, $g_{L}(r)$, of the IR‐Seed2 seed was measured using TLDs and MC simulations were also performed in water and Plexiglas phantoms. Figure 5(a) shows an excellent agreement (within experimental uncertainty) between the measured and calculated values in the Plexiglas phantom. This agreement validates the accuracy of the source and phantom geometry used in the MC simulations in the range of 0.1 cm to 7 cm.

The 2D anisotropy function, F(r,θ), of the IR‐Seed2 was measured and calculated. The measured F(r,θ) for this source in Plexiglas is in good agreement (within experimental uncertainty) with the calculated values in the same medium (Fig. 6). The discrepancy between the measured and calculated anisotropy functions at different distances of 2 cm, 3 cm, and 5 cm is within experimental uncertainty (±7%). Figure 8 displays a discrepancy of ±7% between the MC calculated anisotropy function of the IR‐Seed2 source to water in water with the published data of the 6702 I125, IBt Model 1251L, and Mills Biopharmaceuticals (Mills Biopharmaceuticals Inc., Oklahoma City, OK) model SL‐125/SH‐125 I125 sources at a distance of 5 cm.^3^, ^15^ This figure indicates larger anisotropy functions at small angles for the International Brachytherapy InterSource125, which can be attributed to the absence of end caps on that source model. The values of the calculated and measured anisotropy functions, and anisotropy factors for the IR‐Seed2 source are listed in Tables 4 and 5.

These results show that the higher discrepancies between the calculated and measured values are related to those points which are located in the longitudinal plane of the seed. These discrepancies are due to self‐adsorption and oblique filtration of the radiation in the encapsulating material. The IR‐Seed2 has thicker end caps than the other listed sources in this project.

Overall, the discrepancies between the calculated and measured values of g(r) and F(r,θ) in the distances close to the seed are due to the high gradient dose and relatively large dimensions of TLDs compared to the phantom and seed sizes.

In IR‐Seed2 I125 source, the radioactive material is nonuniformly adsorbed on the surfaces of the resin beads, unlike the other commercially available brachytherapy sources which have been distributed uniformly. Ignoring this property, this source can be used clinically for catheter‐based brachytherapy applications.

## V. CONCLUSION

The dosimetric parameters of the Model IR‐Seed2 I125 source have been determined experimentally and theoretically based on the TG‐43U1 recommendations.^3^ A complete set of both measured and calculated data was presented herein for this source in water and Plexiglas phantoms.

The MC simulation results to water in water are recommended for clinical application of this source model. The data of IR‐Seed2 are comparable to other commercially available I125 brachytherapy sources. Based on these acceptable results, the IR‐Seed2 source is being used just for eye plaque and brain implants that are catheter‐ or applicator‐based implants for their localization.

The design of this source is in progress in terms of X‐ray marker use, and of its application in prostate brachytherapy implantation.

## ACKNOWLEDGMENTS

The authors would like to thank the staff of Radiotherapy Physics Department and Cancer Research Centre for their assistance in performing this project.

## COPYRIGHT

This work is licensed under a Creative Commons Attribution 3.0 Unported License.

## Supporting information

Supplementary Material
